# Chia seed-mediated fabrication of ZnO/Ag/Ag_2_O nanocomposites: structural, antioxidant, anticancer, and wound healing studies

**DOI:** 10.3389/fchem.2024.1405385

**Published:** 2024-07-11

**Authors:** Aisha Rafique, Fatima Amjad, Muhammad Ramzan Saeed Ashraf Janjua, Syed Ali Raza Naqvi, Sadaf Ul Hassan, Hanzla Abdullah, Muhammad Shahid Nazir, Zulfiqar Ali, Abdulaziz A. Alshihri, Maha Abdullah Momenah, Adel Abo Mansour, Majed A. Bajaber, Ahlam A. Alalwiat

**Affiliations:** ^1^ Department of Chemistry, Government College University Faisalabad, Faisalabad, Pakistan; ^2^ Department of Chemistry, COMSATS University Islamabad, Lahore, Pakistan; ^3^ Department of Radiological Sciences, College of Applied Medical Sciences, King Khalid University, Abha, Saudi Arabia; ^4^ Department of Biology, College of Science, Princess Nourah bint Abdulrahman University, Riyadh, Saudi Arabia; ^5^ Department of Clinical Laboratory Sciences, College of Applied Medical Sciences, King Khalid University, Abha, Saudi Arabia; ^6^ Chemistry Department, Faculty of Science, King Khalid University, Abha, Saudi Arabia

**Keywords:** metallic nanoparticles and nanocomposite, green synthesis, antimicrobial *in vitro*, anticancer *in vitro*, wound healing *in vivo*

## Abstract

Plant extract-mediated fabrication of metal nanocomposites is used in cell proliferation inhibition and topical wound treatment, demonstrating significant effectiveness. *Salvia hispanica* L. (chia) seed extract (CE) is used as the reaction medium for the green fabrication of ecofriendly ZnO_(CE)_ nanoparticles (NPs) and Ag/Ag_2_O_(CE)_ and ZnO/Ag/Ag_2_O_(CE)_ nanocomposites. The resultant nanoparticles and nanocomposite materials were characterized using UV–visible, Fourier-transform infrared (FTIR) spectroscopy, scanning electron microscopy (SEM), X-ray diffraction (XRD), and energy-dispersive X-ray (EDX) techniques. In the context of antioxidant studies, ZnO/Ag/Ag_2_O_(CE)_ exhibited 57% reducing power and 86% 2,2, diphenyl-1-picrylhydrazyl (DPPH) free radical scavenging. All three materials showed strong antibacterial activity against *Staphylococcus aureus* (*S. aureus*), *Escherichia coli* (*E.coli*), and *Bacillus subtilis* (*B. subtilis*) bacterial strains. Additionally, ZnO_(CE)_, Ag/Ag_2_O_(CE)_, and ZnO/Ag/Ag_2_O_(CE)_ also revealed 64.47%, 42.56%, and 75.27% *in vitro* Michigan Cancer Foundation-7 (MCF7) cancer cell line inhibition, respectively, at a concentration of 100 μg/mL. Selectively, the most effective composite material, ZnO/Ag/Ag_2_O_(CE)_, was used to evaluate *in vivo* wound healing potential in rat models. The study revealed 96% wound closure in 10 days, which was quite rapid healing compared to wound healing using clinically available ointment. Therefore, in conclusion, the ZnO/Ag/Ag_2_O_(CE)_ nanocomposite material could be considered for further testing and formulation as a good anticancer and wound healing agent.

## 1 Introduction

The unique attributes of nanoparticles are augmented by their higher surface area/active mass-to-weight ratio, reactivity, and bioavailability. Their integration into nanocomposites and matrixes yielded nanomaterials with a unique set of physical, chemical, and biochemical properties, which resulted in intensive studies for a wide range of applications (Bilal et al., 2022). Over the last couple of decades, nanomaterials in the form of nanopowders, nanocomposites, nanofibers, etc*.*, have been under extensive study to be used in potential applications, such as medical products and devices (Haleem et al., 2023), water treatment systems (Naseem and Durrani, 2021), air filters and air purifiers (Mahmoudi et al., 2023), polishing agents (Amir et al., 2022), catalysts (Patra et al., 2023), biosensors (Malik et al., 2023), food packaging and food protection films (Cushen et al., 2014), paints and varnish (Ganguli and Chaudhuri, 2021), and cosmetics (Włodarczyk and Kwarciak-Kozłowska, 2021). Among these applications, the design of innovative and multifunctional nanocomposites for anticancer, antimicrobial, and wound healing agents is of basic interest due to their life-saving nature. The most life-threatening disease is cancer, the most prevalent and leading cause of mortality in the world. Multiple options are in practice to create a barrier to its proliferation in the human body, but none has been perfected yet. Nanomaterials are engineered for this purpose (Nirmala et al., 2023). For example, the treatment of breast cancer involves loading doxorubicin into liposomes, followed by loading siRNA molecules onto solid lipid and other nanoparticles. These advancements were aimed at targeted therapy to enhance therapeutic efficacy (Senapati et al., 2018). Photothermal therapy (PTT), using gold and silver nanoparticles, is being exercised to destroy cancer cells and stimulate immune responses (Borzęcka et al., 2022). Moreover, as drug delivery systems (DDSs), nanoparticles play a pivotal role in improving the therapeutic efficacy of anticancer treatment and reducing the cytotoxic effect (Sharma et al., 2023). Bacterial infection is a global burden due to its life-threatening outbreaks. A long history of microbial infection treatment is associated with antibiotics, but according to the reported data and experienced clinicians, sooner or later, bacterial resistance becomes a hallmark of the use of antibiotics, which reduces their efficacy significantly. Nanomaterials paralyze and kill bacteria effectively and also eliminate the chance of bacterial resistance. These potential properties intensify attempts to develop antibacterial nanomaterials to treat different infectious ailments, such as deep-seated infections and wound healing (Gupta et al., 2019; Nandhini et al., 2024). The use of antibacterial compounds is not only limited to the *in vivo* treatment of bacterial infections but is also required for disinfecting healthcare instruments, especially surgical tools. Reported data advocate that gold nanoparticles and quantum dots, using infrared light, play a vital role in disinfecting the instruments from bacteria (Haleem et al., 2023).

Wounds are injuries to the skin tissues caused by external stimuli or trauma, categorized as acute and chronic wounds based on their healing period. Acute wounds take few weeks to heal, while chronic wounds may take several months. Contamination, colonization, and infection are the three main stages in the wound infection continuum (Li et al., 2021). Wound contamination represents the microbes in non-replicating form in an open wound. This stage does not affect the normal inflammatory response or wound healing process. Untreated microbes start replicating into colonies, which further damages the tissue and partially inactivates the inflammatory response. Further migration and replication of microbes deep into the wound bed result in an immune reaction with the characteristics of infection (acute state) (Swanson et al., 2015). The production of the glycocalyx (biofilm) by some microbes at the wound site leads to the development of a protective layer, which makes the wound hard to detect and treat (chronic state) (Malone et al., 2017). Further progression may lead to systemic complications (cellulitis, osteomyelitis, and septicemia) from topical wounds (Li et al., 2021). Chronic wounds affect approximately 20 million people globally, with an economic burden of approximately $31 billion per year for treatment and management (Järbrink et al., 2017; Nussbaum et al., 2018).

The wound healing process is an intricate interaction among wound cells, the components of the extracellular matrix, and biochemical signal pathways. Commonly exercised traditional wound healing treatments are a slow process, which is associated with some typical limitations such as sluggish tissue regeneration, long-term persistence of wound infections, and poor wound closure. This hampers the wound healing process seriously. Nanotechnology has created new research opportunities in the field of wound healing strategies and has made significant advances in cancer therapy and wound healing (Nandhini et al., 2024). Metallic nanoparticles (NPs), such as silver (Ag), gold (Au), and zinc (Zn), have been reported to have promising bactericidal properties and low cytotoxicity, which make them useful for wound healing (Rajendran et al., 2018). Ag NPs aid in the healing of superficial wounds (Solanki et al., 2023) and are often combined with other metals, particularly Au and Zn, to boost their antibacterial potential. Physical and chemical methods of NP synthesis are gradually being replaced by ecofriendly green synthesis methods because of their multiple demerits, such as the release of toxic and harmful chemicals, consumption of a large amount of energy, and use of complex synthesis conditions and equipment (Ying et al., 2022). The green synthesis of ZnO NP/silica gel dressings using *Aloe barbadensis* leaf extract was found effective in healing mouse wounds (Batool et al., 2020). The blend of Ag and Zn NPs has also been used in wound healing, such as the 1% Ag–ZnO/silver oxide (AgO) nanocomposites currently being investigated for the treatment of topical wounds (de Moura et al., 2022), chitosan dressings loaded with Ag and Zn NPs (chitosan/AgO/ZnO) (Li et al., 2010), and chitosan/polyethylene glycol/ZnO/Ag (chitosan/PEG/ZnO/Ag) nanocomposite dressings (Liu and Kim, 2012). Hydrogels containing Ag@ZnO core–shell nanocomposites synthesized from the leaf extract of *Hibiscus sabdariffa* were used for wound healing in a mouse model (Malone et al., 2017).

*Salvia hispanica* L. is a flowering plant belonging to the mint family (Lamiaceae) native to central and southern Mexico. The seeds obtained from this plant are commonly termed chia seeds, which possess a great deal of nutritional and medicinal value. Chia seeds are a potential source for synthesizing nanomaterials as they are readily available, inexpensive, and can easily be processed into different forms (such as gel, powder, and liquid). This easy processability is the main reason for using chia seeds as a source for nanoparticle synthesis. Chia seeds are made up of proteins, lipids, polysaccharides, some phytochemicals, flavonoids, and phenolic compounds. These flavonoids and phenolic acids act as great reducing agents and stabilizing agents for the nanoparticles. These compounds also provide great antioxidant properties to the nanomaterials; hence, they are potential antibacterial and antioxidant materials. Compounds like alpha-linolenic acid reduce inflammation and can inhibit cancer cell growth, and flavonoids and chlorogenic acid help neutralize harmful free radicals in the body, which can protect the body from bacterial attacks, reduce inflammation, and inhibit cancer cell growth. Phytochemicals like quercetin, kaempferol, and caffeic acid have the chemical ability to interfere with cancer cell proliferation, induce apoptosis (programmed cell death), and inhibit angiogenesis (the formation of new blood vessels that supply tumors), hence contributing to anticancer activity. The chemical composition of chia seeds allows easy modification through simple chemical or enzymatic treatments, further expanding their potential applications in nanoparticle synthesis (Reyes-Caudillo et al., 2008). The use of dark chia seed extract (CE) in the synthesis of Ag NPs boosted their antimicrobial activities (Hernández-Morales et al., 2019). The aim of this study is to synthesize ZnO_(CE)_ NPs, Ag/Ag_2_O_(CE)_, and ZnO/Ag/Ag_2_O_(CE)_ nanocomposites by using the aqueous extract of chia seeds as a reducing and stabilizing reagent for the investigation of anticancer, antibacterial, and wound healing potential.

## 2 Materials and methods

### 2.1 Chemicals

Silver nitrate (AgNO_3_), zinc nitrate hexahydrate (Zn(NO_3_)_2_.6H_2_O), sodium hydroxide (NaOH), sodium phosphate, potassium ferricyanide, trichloroacetic acid, ferric chloride, ascorbic acid, 2,2, diphenyl-1-picrylhydrazyl (DPPH), methanol, 3-(4,5-dimethylthiazol-2-yl)-2,5-diphenyl-2H-tetrazolium bromide (MTT), dimethyl sulfoxide, and Mueller Hinton agar (MHA) were obtained from Sigma-Aldrich and used without further purification. Chia seeds were obtained from the local market. All the solutions were prepared in double-distilled deionized water.

### 2.2 Preparation of the chia seed extract

Chia seeds (9.6 g) were cleaned three times using distilled water and dried at room temperature. After drying, the chia seeds were heated in 100 mL of distilled water for 30 min and immediately filtrated using a nylon-fiber sieve. The CE was stored in a refrigerator at 5°C and used for NP synthesis a day after its preparation for the best antimicrobial results (Hernández-Morales et al., 2019).

### 2.3 Synthesis of ZnO NPs

The CE (30 mL) was heated at 60°C, followed by the addition of 1.33 g of Zn(NO_3_)_2_.6H_2_O (0.15 M) and agitated for at least 4 h. The solution color changed from transparent to light brown, indicating the formation of precipitates. The solution was cooled to room temperature and then centrifuged at 4,000 rpm for 20 min. The prepared nanoparticles were rinsed repeatedly in distilled water to remove any unreacted material. The material was dried at 80°C for 6 h and then calcinated at 500°C for 4 h (Sorbiun et al., 2018).

### 2.4 Synthesis of the Ag/Ag_2_O nanocomposite

Ag/Ag_2_O_(CE)_ was synthesized by adding 0.8491 g AgNO_3_ to 20 mL deionized water and mixing well to form a uniform solution (0.25 M), followed by the addition of 1.6 mL CE. The mixture was stirred vigorously for 15 min. The precipitates turned the color of the solution from transparent to light yellow. Further overnight stirring at room temperature was carried out to obtain AgO NPs. The resultant mixture was centrifuged at 4,000 rpm for 10 min. The prepared precipitates were separated, dried at 80°C, and then calcinated at 300°C for 2 h. The resulting brown precipitates indicated the successful synthesis of Ag/Ag_2_O_(CE)_ (Hernández-Morales et al., 2019).

### 2.5 Synthesis of the ZnO/Ag/Ag_2_O nanocomposite

The ZnO/Ag/Ag_2_O nanocomposite was prepared using a co-precipitation method with slight modifications (Hernández-Morales et al., 2019). The nanocomposite was synthesized by the addition of AgNO_3_ (0.212 g∼0.025 M [0.0012 mol]) and Zn(NO_3_)_2_.6H_2_O (0.376 g∼0.025 M [0.0012 mol]) to 50 mL of distilled water. The components were mixed well to create a homogeneous solution. Then, 1.6 mL of CE was added dropwise under vigorous stirring for 15 min at ambient temperature. The change in the color of the solution from transparent to brown was an indication of precipitate formation. Overnight stirring of the solution resulted in brown precipitates settling at the bottom of the beaker. The mixture was centrifuged at 4,000 rpm for 10 min, followed by the collection of precipitates, drying at 80°C for 6 h, and then calcination at 400°C for 4 h. ZnO/Ag/Ag_2_O_(CE)_ nanocomposites were consequently obtained in the form of brown powder. The visual analysis of the final nanomaterials synthesized is shown in Supplementary Figure S1.

### 2.6 Characterizations

A UV–visible spectrophotometer (Shimadzu UV-2550 Spectrophotometer), Fourier-transform infrared (FTIR) spectrophotometer (Nicolet Magna 550 Spectrophotometer), scanning electron microscope, powder X-ray diffractometer, and energy-dispersive X-ray (EDX) spectrophotometer were used to characterize ZnO_(CE)_ NPs and Ag/Ag_2_O_(CE)_ and ZnO/Ag/Ag_2_O_(CE)_ composites in order to identify their physical features. The UV–visible spectra in the range of 200–800 nm were recorded. The FTIR spectrophotometer covering a wave number range of 650–4,000 cm^-1^ was utilized to determine the functional groups found in ZnO_(CE)_ NPs and Ag/Ag_2_O_(CE)_ and ZnO/Ag/Ag_2_O_(CE)_ nanocomposites. SEM investigated the appearance and structure of nanoparticles and nanocomposites. XRD patterns were recorded using a PANalytical X’Pert (Naseem and Durrani, 2021) material research diffractometer with CuKα radiation to determine sample phases in the range of 10°–80°.

### 2.7 Antioxidant study

The antioxidant potential of the synthesized NPs and nanocomposites was determined using the most commonly adopted methods, such as reducing power and DPPH free radical scavenging assays.

#### 2.7.1 Reducing power assay

The antioxidant activity of ZnO_(CE)_, Ag/Ag_2_O_(CE)_, and ZnO/Ag/Ag_2_O_(CE)_ was determined according to a method described previously (Soni and Dhiman, 2017). In brief, 2.5 mL of different concentrations (62.5 μg/mL, 125 μg/mL, 250 μg/mL, 500 μg/mL, and 1,000 μg/mL) of the sample (ZnO_(CE)_ or Ag/Ag_2_O_(CE)_ or ZnO/Ag/Ag_2_O_(CE)_) was added separately to 2.5 mL of 200 mmol/L sodium phosphate (pH 6.6), which was then mixed with 2.5 mL of the potassium ferricyanide solution. The mixture was then incubated at 50 C for 20 min, followed by the addition of 2.5 mL of 10% trichloroacetic acid (w/v), and centrifuged at 650 rpm for 10 min. After centrifugation, 5 mL of the supernatant was mixed with 5 mL of distilled water and 1 mL of 0.1% ferric chloride. The absorbance of this solution was measured at 700 nm compared to ascorbic acid used as the standard. Higher absorbance yielded a higher reducing potential.

#### 2.7.2 DPPH free radical scavenging assay

The (DPPH free radical assay was carried out to assess the radical scavenging activity of the antioxidant component using a previously reported protocol (Al-Shmgani et al., 2016). In brief, a ZnO_(CE)_, Ag/Ag_2_O_(CE)_, or ZnO/Ag/Ag_2_O_(CE)_ sample methanol solution of different concentrations (125 µg–1,000 μg/mL) was mixed with 4 mL of the 0.2 mM methanol solution of DPPH free radical. After 30 min of incubation at room temperature, the absorbance was recorded at 517 nm. The scavenging activity was measured using Eq 1:

$$\%\text{ of inhibition}=\left(A_{\text{blank}}\;–\;A_{\text{Sample}}\right)/A_{\text{blank}}*100,$$ (1)

where A_blank_ is the absorbance of the DPPH solution and A_sample_ is the absorbance of the sample solution. The lower the absorbance, the higher the DPPH radical scavenging activity.

### 2.8 Antibacterial potential study

The antimicrobial activity of ZnO_(CE)_ NPs and Ag/Ag_2_O_(CE)_ and ZnO/Ag/Ag_2_O_(CE)_ nanocomposites was investigated using the well diffusion method with slight modifications, as previously reported (Al-Shmgani et al., 2016). The antibacterial activity was assessed against three bacterial strains: *S. aureus*, *B. subtilis*, and *E. coli*. An agar solution containing 3.8 gMH of agar was prepared and sterilized in an autoclave. The sterilized media were then transferred to Petri dishes, and bacterial cultures were spread on the solid medium surface. A stock solution of nanocomposites was prepared at various concentrations (3 mg/mL, 1.5 mg/mL, 0.750 mg/mL, and 0.375 mg/mL). DMSO and CFR 30 of 30 μg/mL were used as the negative and positive controls, respectively. Six wells were punctured in each Petri plate, 40 ppm solution from each dilution was inoculated into every Petri dish, and a CFR 30 disc was placed in the center. These Petri dishes were incubated at 37°C for 24 h. After 24 h, the nanocomposites inhibited the growth of the bacterial cultures, and the diameter of the zone of inhibition (ZOI, mm) was measured against each organism. Triplicate measurements were taken to ensure the accuracy and reliability of the results.

### 2.9 Anticancer activity

Using a slightly altered MTT assay developed by Bio Basic Inc. (Canada), the cytotoxicity of ZnO_(CE)_ NPs and Ag/Ag_2_O_(CE)_ and ZnO/Ag/Ag_2_O_(CE)_ nanocomposites was evaluated using the breast cancer cell line MCF7 provided by the American Type Culture Collection (ATCC) center (Hashem and El-Sayyad, 2023). The viability and inhibition percentages were determined using the following Eqs 2, 3, respectively:

$$Viability\%=\frac{Sample\;optical\;density}{Control\;optical\;density}\times 100$$ (2)

%Inhibition=100−Viability% (3)

### 2.10 Wound healing *in vivo* studies

The Department of Pharmaceutics, Government College University Faisalabad, Pakistan, approved the experimental methods for analyzing wound healing. The four mice used in the experiment were categorized into two groups, each with a pair of mice. Group I was designated as the control group, and group II was treated with ZnO/Ag/Ag_2_O_(CE)_. Under anesthesia with chloroform, a full-thickness excision wound measuring 6 mm was made after shaving the dorsal side hair of the mice using a sterile surgical blade. The mice in the treatment group were given 1 mL of synthetic ZnO/Ag/Ag_2_O_(CE)_ (2 mM) in the form of niosomes, whereas the mice in the control group were given 1 mL of standard saline. These ingredients were mixed into a 2 × 2-cm^2^ dressing, which was then placed on the wound bed. For 10 days, this application was done once every day. The contraction of the wound tissue was observed at intervals of 0, 2, 4, 6, 8, and 10 days to gauge the degree of wound healing. Additionally, on the specified days, pictures of the wounds in the control and treated mice were taken and compared (Al-Shmgani et al., 2016).

## 3 Results and discussion

### 3.1 FTIR studies

**FIGURE 1:**
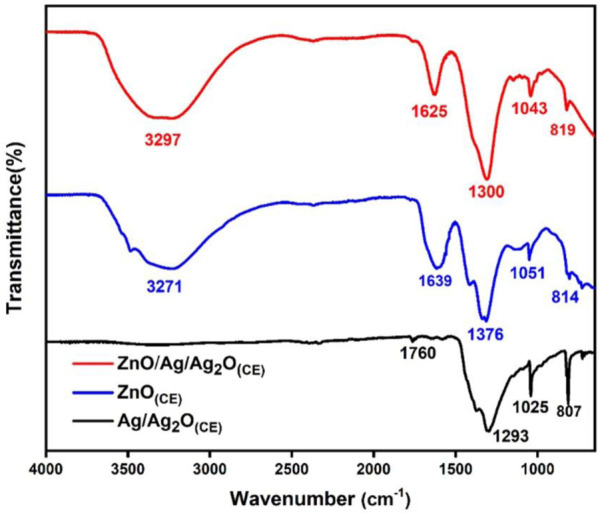
FTIR spectra of ZnO_(CE)_, Ag/Ag_2_O_(CE)_, ZnO/Ag/Ag_2_O_(CE)_.

FTIR analysis identified the functional groups in the ZnO_(CE)_ NPs and Ag/Ag_2_O_(CE)_, and ZnO/Ag/Ag_2_O_(CE)_ nanocomposites, as shown in Figure 1, with spectral ranges from 4,000 to 600 cm^−1^. Specifically, the band within 3,483.2–3,286.0 cm^−1^ is due to O-H group stretching vibrations from phytochemicals like polyphenols, alkaloids, and flavonoids in the chia seed extract, confirming the existence of diverse encapsulated and reducing bioactive molecules in the extract (Hernández-Morales et al., 2019). In the case of Ag/Ag_2_O_(CE)_, the faint band observed in the AgO spectrum at approximately 3,238 cm^−1^ can be allocated to the stretching of the C–H bonds in the aldehyde molecule. Various absorbent bands at 1,760 cm^–1^, 1,373 cm^–1^, 1,293 cm^−1^, and 1,070–1,000 cm^−1^ are due to C=C bond vibrations associated with stretching (Laouini et al., 2021), the presence of C-O (Sobhani-Nasab and Behpour, 2015) and C=O stretching vibrations due to carbonyl and carboxylate groups (Jaiswal et al., 2022), and CH_2_O stretching or bending vibrational bands (Chen et al., 2014). Furthermore, C-H bending was identified as the cause of the band observed at 807 cm^–1^ (Barwant et al., 2022). The absorption range observed in ZnO_(CE)_ at 3,271 cm^−1^, 1,638.7 cm^−1^, 1,376.2 cm^–1^, 1,050.9 cm^−1^, and 814 cm^–1^ is linked to the O-H stretching vibrations of alcoholic and carboxylic groups, C=C bonding in alkene, C-H stretching of the methyl group, C-O bonding in polysaccharides (Zafar et al., 2021), and the O-H functional group (Sorbiun et al., 2018). The FTIR spectrum of ZnO/Ag/Ag_2_O_(CE)_ indicates the existence of a strong band at approximately 3,296 cm^-1^ linked to the vibratory stretching of the hydrogen-bonded O-H group, indicating that the water molecules are captured by the samples (Ding et al., 2019). The bands observed at 1,625 cm^–1^ and 1,524 cm^–1^ indicated the C=C stretching vibrations (Thatikayala et al., 2020) and the N-O stretching band, respectively. Furthermore, bands between 1,300 and 1,043 cm^–1^ were detected, attributed to the O-H bending and CO-O-CO stretching of bonds. C = C bending vibrations of unsaturated compounds in the seed extract were observed at 819 cm^–1^. In the case of ZnO/Ag/Ag_2_O_(CE)_, a distinct peak was observed within the 1,094–821 cm^−1^ range, corresponding to the combined absorptions of Zn-O and Ag-O bonds.

### 3.2 XRD studies

The X-ray diffraction spectrum of Ag/Ag_2_O_(CE)_ is shown in Supplementary Figure S2. Several diffraction patterns were observed in the face-centered cubic structure at 38.1, 44.3, and 64.6 corresponding to the metallic Ag (111), (200), and (220), respectively (JCPDS card no. 04-0783) (Elyamny et al., 2021). Changes required. Peaks at 32.7°, 38.06°, 54.8°, and 65.6° were assigned to cubic phase Ag_2_O diffraction planes (111), (200), (220), and (311) (JCPDS card no. 41-1104) (Sophia et al., 2020). The average size of the Ag/Ag_2_O nanoparticles was calculated using Scherrer’s equation (Eq. 4) and the full breadth at half-maximum (FWHM) of the diffraction patterns.

$$D=\frac{K\lambda }{\beta \cos\theta }.$$ (4)

Here, *D* is the crystalline size of the nanoparticles, *K* denotes a constant of 0.94, λ is the wavelength of radiation (1.54056 Å for CuKα radiation), β is the peak breadth at half-maximum intensity, and *Ѳ* is the peak position. Thus, the average size of the green synthesized Ag/Ag_2_O_(CE)_ nanoparticles was 46.6 nm. The XRD pattern of the green synthesized ZnO nanoparticles, as shown in Supplementary Figure S2, indicates the crystalline structure of the synthesized nanoparticles. The sharp diffraction bands were observed at 2Ѳ values: 31.68, 34.34, 36.02, 47.51, 56.26, 62.67, 67.1 68.18, 69.73, 72.7, and 77.7 of (100), (002), (101), (102), (110), (103), (200), (112), (201), (004), and (202) planes, respectively, which confirms the hexagonal wurtzite structure (JCPDS no. 75-1526) of the synthesized nanoparticles (Althubiti et al., 2023). Thus, the average dimension of green synthesized ZnO nanoparticles is estimated to be 16.15 nm.

**FIGURE 2:**
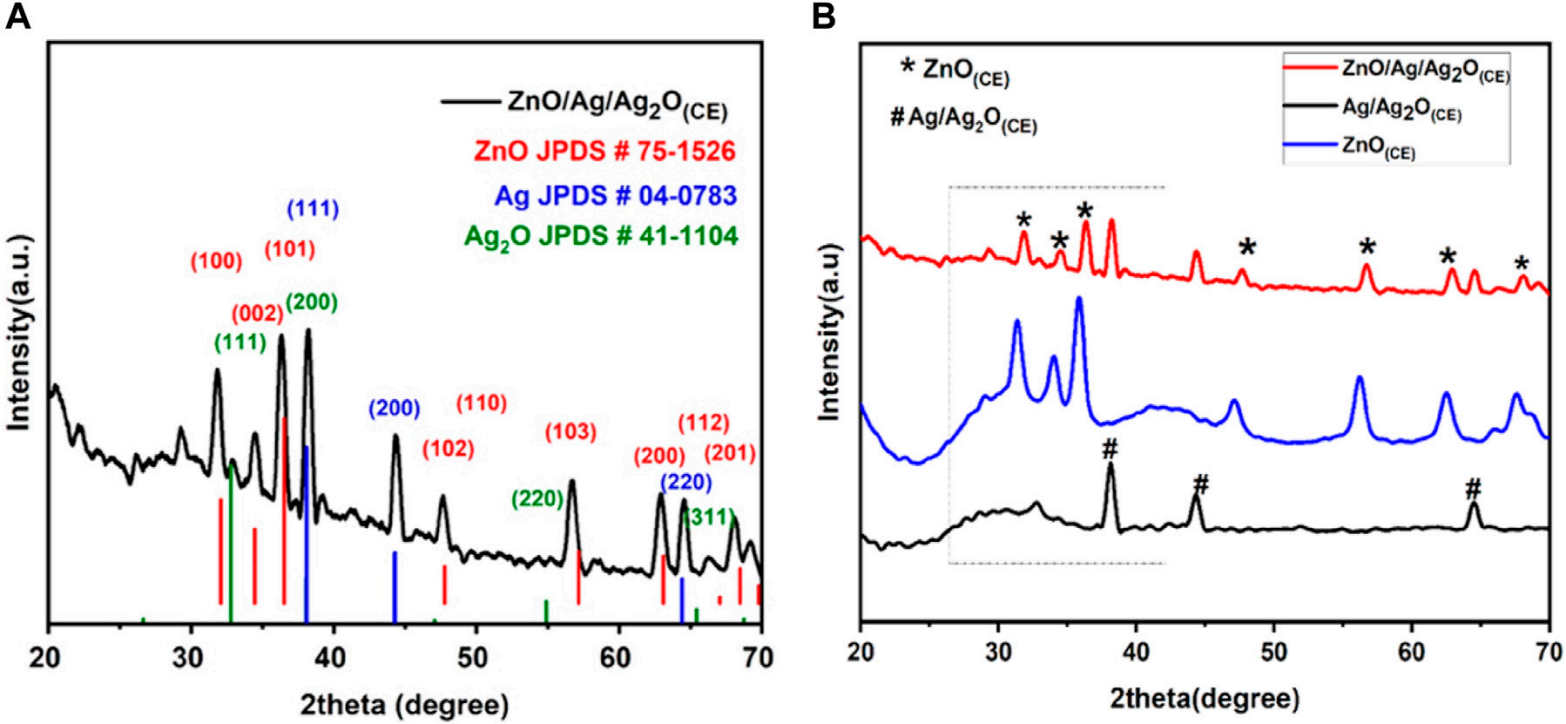
**(A)** XRD spectra of ZnO/Ag/Ag_2_O_(CE)_. **(B)** Stacked XRD graphs of ZnO_(CE)_, Ag/Ag_2_O_(CE),_ and ZnO/Ag/Ag_2_O_(CE)._

The XRD spectra of the ZnO/Ag/Ag_2_O_(CE)_ nanocomposite obtained (Figure 2A) were confirmed against the standards of Ag, Ag_2_O, and ZnO. The diffraction lines located at 38.1 (111), 44.28 (200), and 64.6 (220) verified the presence of Ag in the ZnO/Ag/Ag_2_O composite (JCPDS card no. 04-0783) (Umukoro et al., 2016). Peaks at 32.1 (111), 38.1 (200), 55.1 (220), and 65.4 (311) can be attributed to the crystalline plane of Ag_2_O (JCPDS card no. 41-1104). Thus, it is validated that Ag and Ag_2_O exist together in ZnO/Ag/Ag_2_O (Umukoro et al., 2016). The remaining characteristic peaks at 31.8°, 34.4°, 36.3°, 47.5°, 56.6°, 62.9°, 67.9°, 68.7°, and 69.1° match (100), (002), (101), (102), (110), (103), (200), (112), and (201) crystalline orientations of ZnO (Ding et al., 2019). The suggested average particle size of the nanocomposite was 22.42 nm. The doping of ZnO into Ag/Ag_2_O induces alterations in peak intensity and diffraction angle in the XRD patterns depicted in Figure 2B. This doping may introduce new phases into the host material, leading to reduced peak intensity as crystal planes undergo transformation. Additionally, a minor shift toward higher angles occurs, which is attributed to the expansion of host lattice parameters in the final composite ZnO/Ag/Ag_2_O (Hosseini et al., 2015; Pandi et al., 2023).

### 3.3 UV–visible studies

**FIGURE 3:**
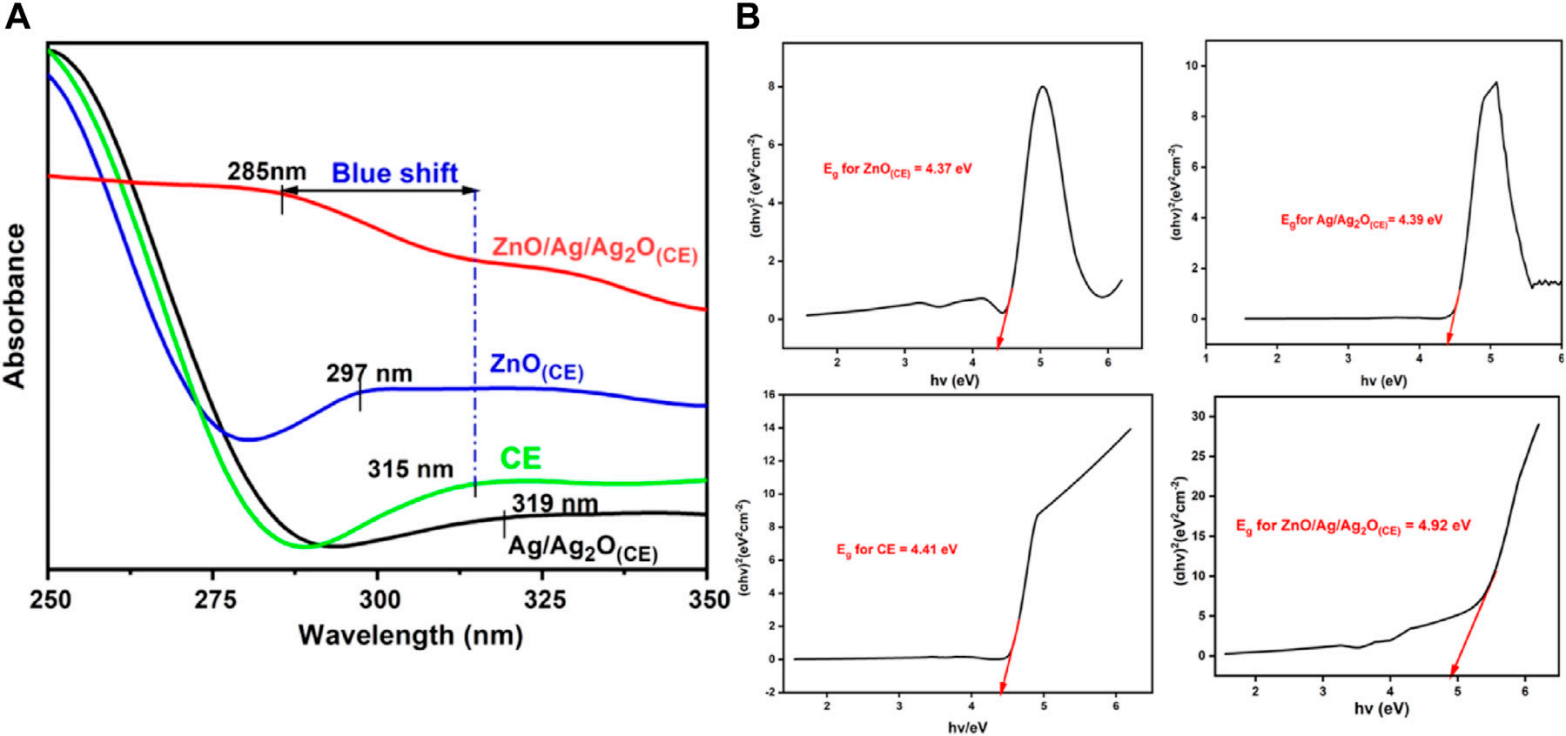
**(A)** UV–visible spectra of chia seed extract, ZnO_(CE)_, Ag/Ag_2_O_(CE)_, and ZnO/Ag/Ag_2_O_(CE)_. **(B)** Tauc’s plot analysis for band gap energy calculations of the prepared nanocomposites.

The UV–visible spectra of chia seed mucilage, ZnO_(CE)_ NPs, and Ag/Ag_2_O_(CE)_ and AgO.ZnO_(CE)_ nanocomposites are shown in Figure 3A. The peak observed at 315 nm shows the interaction of phenols, flavonoids, and alkaloids in the chia seed mucilage. The chia seed mucilage peak is used as the standard. Ag/Ag_2_O_(CE)_ shows a peak at 319 nm, indicating the conjugation of AgO and the chia seed due to the appearance of a redshift, while the ZnO_(CE)_ peak was observed at 297 nm, showing a blue shift compared to the chia seed mucilage. The peak at 285 nm exhibited by ZnO/Ag/Ag_2_O_(CE)_ showed a higher blue shift of 30 nm compared to the chia seed mucilage. This optical property was also supported by calculating the Tauc plots for the Ag/Ag_2_O_(CE)_, ZnO_(CE)_, chia seed extract, and final ZnO/Ag/Ag_2_O_(CE)_ composite (Figure 3B). The band gap energies of Ag/Ag_2_O_(CE),_ ZnO_(CE)_, chia seed extract, and ZnO/Ag/Ag_2_O_(CE)_ were calculated as 4.39, 4.38, 4.41, and 4.92 eV, respectively. This increase in the band gap energy of the final composite compared to the Ag/Ag_2_O_(CE),_ ZnO_(CE)_, and chia seed extract also confirms the predicted blue shift. This increase also justified the infiltration of the dopants in the final composite.

### 3.4 SEM analysis

**FIGURE 4:**
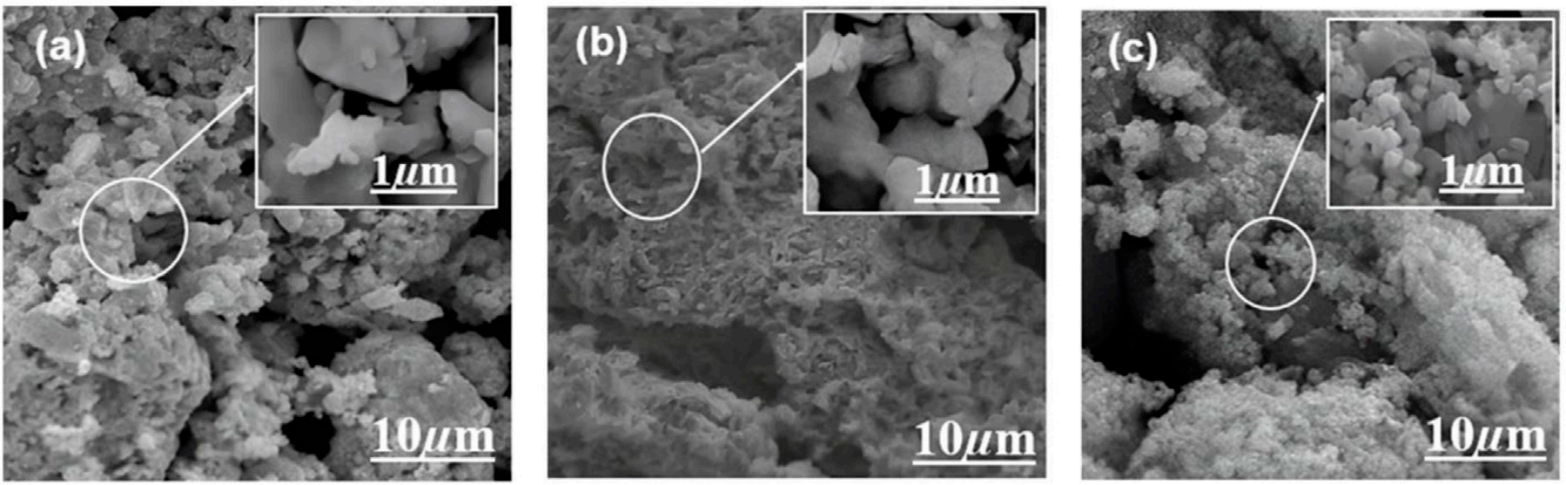
SEM images of **(A)** Ag/Ag_2_O_(CE)_, **(B)** ZnO_(CE)_, and **(C)** ZnO/Ag/Ag_2_O_(CE)_.

SEM is used to study the fabrication of the Ag/Ag_2_O_(CE)_ nanocomposite (Figure 4A) and shows a particle-like heterostructure; there is no obvious difference between the morphology and size of Ag and Ag/Ag_2_O (Yang et al., 2016). Figure 4B shows the SEM images of ZnO_(CE)_ NPs at a lower resolution; it shows that particles are agglomerated and complete separation has probably not happened, while a higher magnification image shows irregularly shaped nanoparticles (Mohan and Renjanadevi, 2016). Figure 4C shows a spherical-shaped nanocomposite of ZnO/Ag/Ag_2_O_(CE)_ (Umukoro et al., 2016). EDX analysis also showed the presence of silver oxide, zinc oxide, and the ZnO/Ag/Ag_2_O composite (Supplementary Figure S3).

### 3.5 Antibacterial studies

Bio-synthesized ZnO NPs and Ag/Ag_2_O and ZnO/Ag/Ag_2_O nanocomposites were evaluated for their antibacterial activity toward *S. aureus*, *B. subtilis*, and *E. coli* using the well diffusion approach, as shown in Supplementary Figure S4. It was observed that microbial growth of 3 mg/mL of NPs showed the highest diameter of the zone of inhibition against each microorganism. The maximum zone of inhibition was shown by Ag/Ag_2_O_(CE)_ at 3 mg/mL against *B. subtilis*, at 17 mm and 14 mm toward *S. aureus*, and 13.5 mm against *E. coli*, while ZnO_(CE)_ demonstrated the maximum ZOI at 16 mm against *B. subtilis*, 14 mm against *S. aureus*, and 12.5 mm against *E. coli*. The ZOI was shown by ZnO/Ag/Ag_2_O_(CE)_ at 25.3 mm against *B. subtilis*, 21 mm against *S. aureus*, and 17.5 mm against *E. coli* at 3 mg/mL.

**FIGURE 5:**
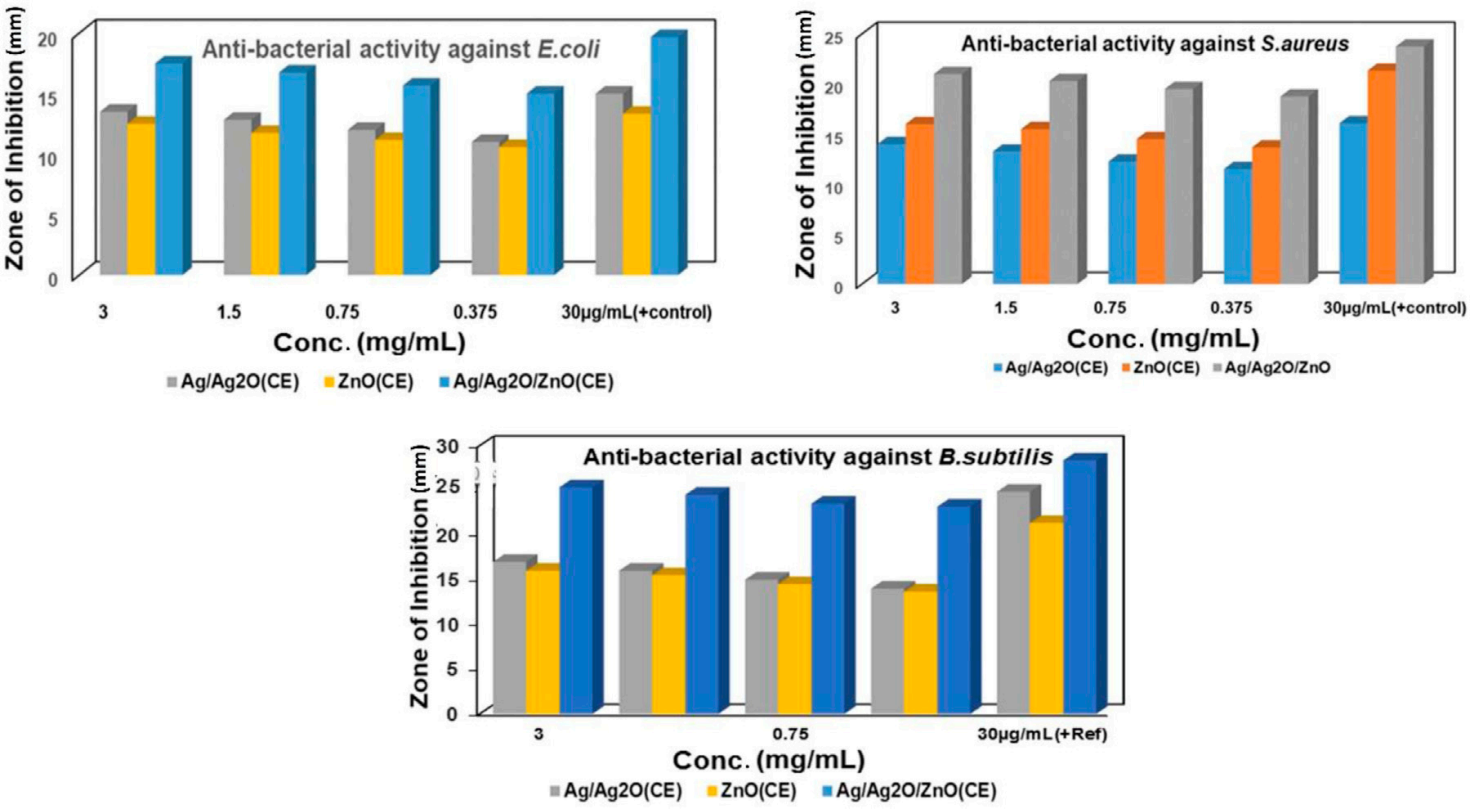
Antibacterial activity of ZnO_(CE)_, Ag/Ag_2_O_(CE)_, and ZnO/Ag/Ag_2_O_(CE)_ against *B. subtilis*.

The negative control showed no ZOI, and the positive control showed the highest inhibition against *B. subtilis*. Among these NPs and nanocomposites, ZnO/Ag/Ag_2_O_(CE)_ exhibited the maximum zone of inhibition against *B. subtilis* at 25 mm. ZnO is toxic to bacteria and has antibacterial properties against resilient species (Bilal et al., 2022). Previous studies showed that growth inhibition increased with increasing NP content in wells, and a similar case was observed in this study (Lawrence, 2015). Depending on the size and concentration of the NPs, the inhibitory zone dimension differed against various bacteria. Antimicrobial activity against *S. aureus, E. coli*, and *B. subtilis* is shown in Figure 5.

The general antibacterial action of the prepared nanocomposites occurs when ZnO and Ag/Ag_2_O nanoparticles work synergistically for their antibacterial activity. ZnO nanoparticles generate reactive oxygen species (ROS), such as superoxide radicals (O•^2−^), hydroxyl radicals (OH•), and singlet oxygen, under light irradiation or in an aerobic environment. The reaction can be represented as

$$\text{ZnO}+H_{2}O+O_{2}\to {\text{Zn}}^{2+}+2\text{OH}-+O{•}^{2-}.$$

These ROS can induce oxidative stress in bacterial cells, causing damage to lipids, proteins, and DNA, ultimately causing cell death. Similarly, Ag/Ag_2_O nanoparticles release silver ions (Ag+) in the presence of aqueous media or in contact with bacterial cell membranes. The reaction can be represented as

$$\text{Ag}+H_{2}O⇌\text{Ag}++\text{OH}-.$$

**FIGURE 6:**
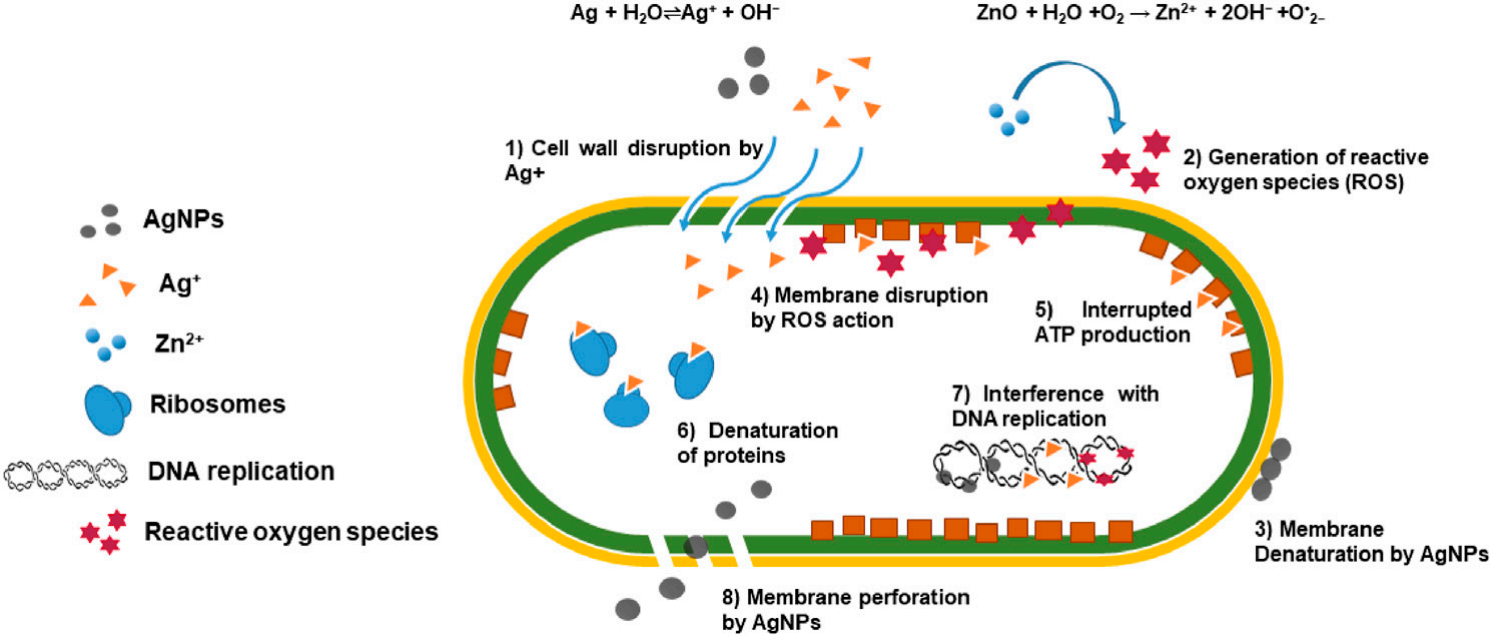
Antibacterial action of the prepared ZnO/Ag/Ag_2_O nanocomposites.

Silver ions disrupt bacterial cell membrane integrity, interfere with cell division, and inhibit enzymatic activity crucial for bacterial survival. This combined action of ROS generated by ZnO and silver ions released from Ag/Ag_2_O nanoparticles results in a synergistic antibacterial effect. ROS cause oxidative damage to bacterial cells, making them more susceptible to the antimicrobial action of silver ions. Additionally, silver ions can enhance ROS generation by ZnO through redox reactions, further amplifying the antibacterial activity. The cumulative effects of ROS-induced oxidative stress and silver ion toxicity lead to irreversible damage to bacterial cells. This antibacterial action is shown in Figure 6.

### 3.6 Antioxidant activities

#### 3.6.1 Reducing power activity

**FIGURE 7:**
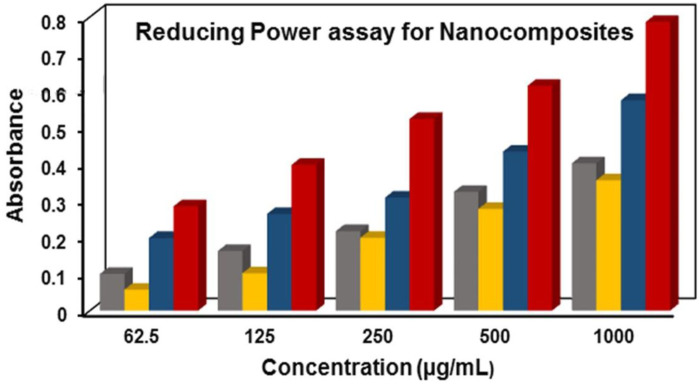
Reducing power assay for ZnO_(CE)_ (yellow), Ag/Ag_2_O_(CE)_ (gray), ZnO/Ag/Ag_2_O_(CE)_ (blue) and ascorbic acid_(Ref)_ (red).

Free radicals which are highly reactive molecules can be neutralized by obtaining electrons from antioxidants. Antioxidants stabilize these free radicals by donating electrons, known as their reducing power. This reducing power is utilized in the chemical reaction where Fe^+3^/ferrocyanide complex is converted to its ferrous(Fe^+2^) form through direct electron donation (Karthick et al., 2012). The product was measured at 700 nm after a strong Prussian blue complex was formed. The reducing capacity of Ag/Ag_2_O_(CE)_ exhibited absorbance values of 0.099, 0.162, 0.216, 0.323, and 0.401, whereas ZnO_(CE)_ displayed absorbance values of 0.0571, 0.1016, 0.198, 0.277, and 0.355. The ZnO/Ag/Ag_2_O_(CE)_ nanocomposite exhibited absorbance values of 0.197, 0.263, 0.307, 0.433, and 0.573, while the standard ascorbic acid exhibited optical density values of 0.284, 0.397, 0.522, 0.612, and 0.786, as shown in Figure 7. Increased absorbance of the reaction mixture or concentration-dependent reducing power was demonstrated by the nanocomposites. However, their reducing power was not as powerful as that of ascorbic acid (standard), which demonstrated the highest reducing activity. Reactive radicals can be reduced to more stable and non-reactive forms by receiving electrons from antioxidant substances (Gülçın et al., 2003). The outcomes from such investigations indicated that the synthesized ZnO/Ag/Ag_2_O_(CE)_ possessed higher antioxidant activity than Ag/Ag_2_O_(CE)_ and ZnO_(CE)_. It was found that absorbance increased with the sample concentration. Higher reaction mixture absorbance suggests higher reduction efficiency.

#### 3.6.2 DPPH free radical scavenging study

**FIGURE 8:**
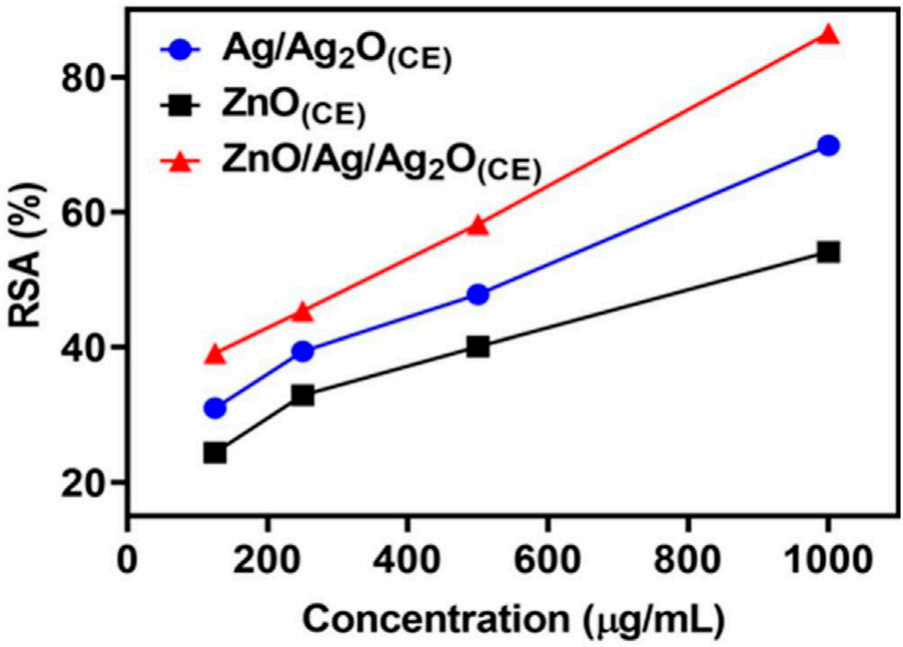
DPPH radical scavenging activity of Ag/Ag_2_O_(CE)_, ZnO_(CE)_, and ZnO/Ag/Ag_2_O_(CE)_.

The free radical scavenging activity of the ZnO_(CE)_ NPs and Ag/Ag_2_O _(CE)_ and ZnO/Ag/Ag_2_O_(CE)_ nanocomposites was evaluated using the DPPH technique. The analysis was carried out thrice. At the maximum concentration of 1,000 μg/mL, the synthesized Ag/Ag_2_O _(CE)_ had better antioxidant activity of 69.91%, whereas ZnO _(CE)_ exhibited higher DPPH activity of 52.138% at 1,000 μg/mL. ZnO/Ag/Ag_2_O _(CE)_ showed the highest radical scavenging activity of 86.554% at 1,000 μg/mL (Aththanayaka et al., 2023). Antioxidant activity increased with the concentration of the tested samples in the DPPH assay (Awan et al., 2023). Thus, the antioxidant activity of ZnO/Ag/Ag_2_O _(CE)_ was higher than that of Ag/Ag_2_O _(CE)_ NPs and ZnO_(CE)_, which was significant (Figure 8).

### 3.7 Anticancer activity

**FIGURE 9:**
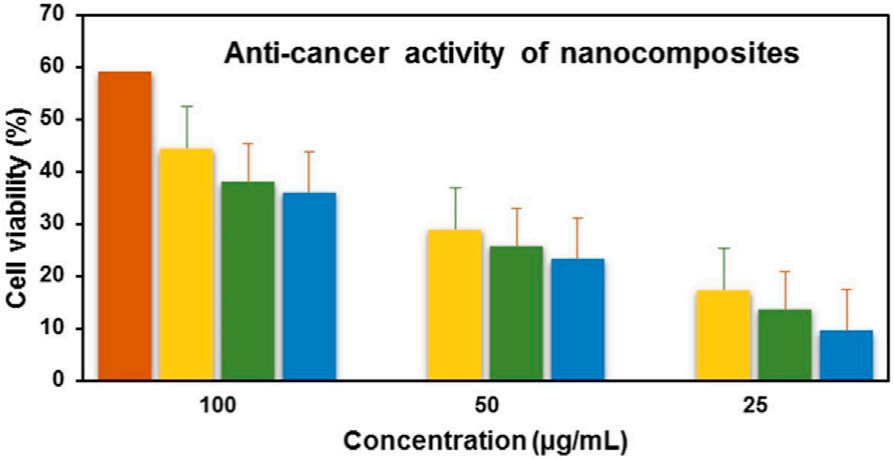
Anticancer activity of doxorubicin(+) (red), ZnO_(CE)_ (green), Ag/Ag_2_O_(CE)_ (blue), ZnO/Ag/Ag_2_O (yellow).

**FIGURE 10:**
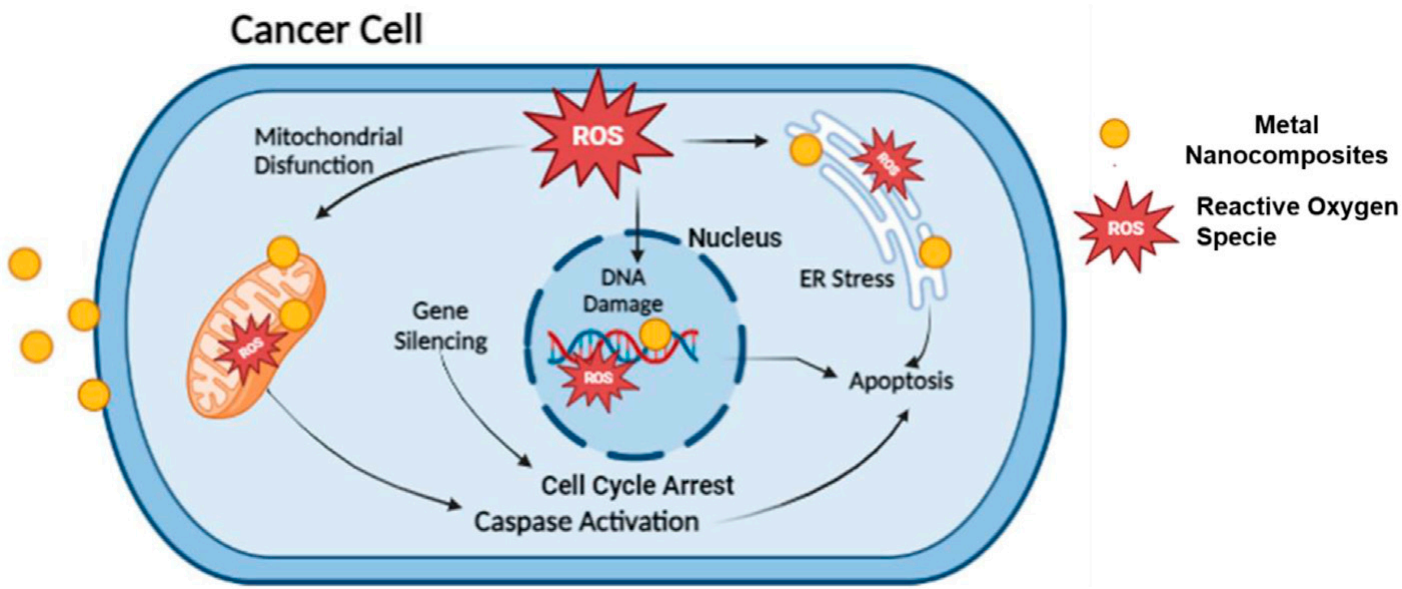
Proposed anticancer activity mechanism of the ZnO/Ag/Ag_2_O nanocomposite.

Anticancer analysis results revealed that ZnO_(CE)_ NPs and Ag/Ag_2_O_(CE)_ and ZnO/Ag/Ag_2_O_(CE)_ nanocomposites exhibited anticancer activity against MCF7 breast cancer cells, with IC_50_ values of 100.24, 100.26, and 100.23, respectively (Narayan et al., 2010). The anticancer activity of ZnO_(CE)_, Ag/Ag_2_O_(CE)_, and Zn/Ag/Ag_2_O_(CE)_ toward MCF7 at a concentration of 100 μg/mL was 64.47%, 42.56%, and 75.27%, respectively, as shown in Figure 9. Metal oxide nanoparticles (ZnO and Ag/Ag_2_O) and nanocomposites (ZnO/Ag/Ag_2_O) generate free radicals, which give rise to ROS. The ROS induce oxidative stress in cancer cells by disrupting the cellular metabolic pathways and damaging DNA, proteins, and lipids, leading to apoptosis. In the ZnO/Ag/Ag_2_O nanocomposite, zinc and silver free radicals interfere with the mitochondrial protease, resulting in apoptosis through caspase activation, and inhibit the cell cycle to stop further cancer proliferation (Roshani et al., 2023). The proposed anticancer mechanism of the ZnO/Ag/Ag_2_O nanocomposite is shown in Figure 10.

### 3.8 *In vivo* wound healing studies

An excision wound model was used to illustrate the wound healing ability of the synthesized Ag NPs. During therapy, ZnO/Ag/Ag_2_O_(CE)_-treated wounds showed no indication of microbial contamination, hemorrhage, or pus development, whereas the control wounds revealed significant inflammation and bleeding. From day 6 onwards, the ZnO/Ag/Ag_2_O_(CE)_-treated group showed distinguished wound closure and decreased wound size, which improved during the remaining days of treatment compared to controls (Supplementary Figure S5).

**FIGURE 11:**
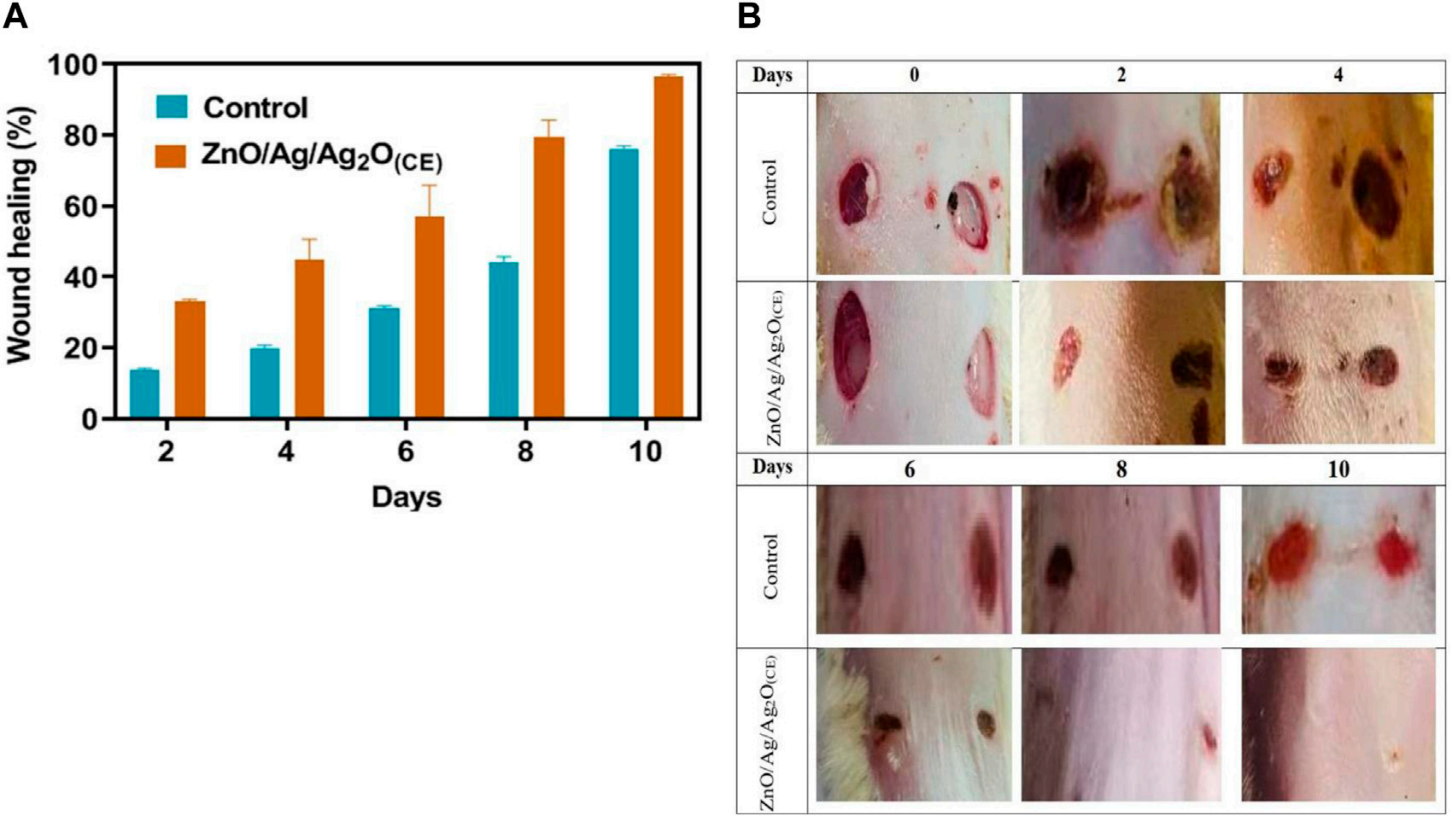
Quantitative **(A)** and visual **(B)** analysis of wound healing in rats.

The ZnO/Ag/Ag_2_O_(CE)_-treated wound had approximately 96% closure at the conclusion of the trial, whereas the control wound had approximately 76% closure (Figure 11A). A visual analysis (Figure 11B) showed that ZnO/Ag/Ag_2_O_(CE)_-treated rats had superior wound healing activity than control groups. Previous research has demonstrated the possible influence of AgNPs on wound healing in an animal species and demonstrated that quick healing and improved cosmetic quality appear in a dose-dependent manner. Moreover, AgNPs showed advantages due to their antimicrobial properties, mitigation of wound swelling via decreased lymphocyte and mast cell intrusion, and amendment of fibrogenic cytokines (Tian et al., 2006). Similarly, the impact of AgNPs on dermal stiffness and epidermis re-epithelialization during wound recovery indicated that AgNPs may speed up wound closure (Liu et al., 2010). This characteristic is considered to enhance keratinocyte proliferation and movement.

In addition, AgNPs may promote the differentiation of fibroblasts into myofibroblasts, resulting in wound contraction (Gunasekaran et al., 2011). Zinc oxide is valuable in wound dressings because of its capability to protect against bacteria (Shu et al., 2023). The enhanced wound healing efficacy results primarily from the synergistic antimicrobial effects of Ag and Zn, leading to a reduction in inflammation. This not only prevents additional infections in the wound but also accelerates the epithelialization process by creating a favorable healing environment (Kantipudi et al., 2018).

Peng, Y. et al. (2022) demonstrated the preparation of cellulose/chitosan/Ag/Ag_2_O/ZnO nanocomposites through synthetic methods, evaluating their efficacy against *E. coli* and *S. aureus* bacterial strains (Peng et al., 2022). Aththanayaka, S. et al. (2023) outlined a synthetic approach for synthesizing Ag/Ag_2_O/ZnO nanocomposites utilizing Murusi peel and Kew peel, showcasing promising antioxidant activity through DPPH radical scavenging assays (Aththanayaka et al., 2023). Hezam, A. et al. (2023) attributed the antibacterial activity of Ag, ZnO, and Ag_2_O to intrinsic properties while also investigating the cytotoxicity of Ag/Ag_2_O/ZnO on Huh-7 human liver cancer cells, yielding IC_50_ values of 112.9 and 313 μg/mL for samples prepared using 3 mL (3AgZn) and 7 mL AgNO_3_ (7AgZn), respectively (Hezam et al., 2023). Ramakrishnegowda, D. H. et al. (2023) reported potent antibacterial activity against *E. coli*, *S. aureus*, and *B. subtilis*, with higher efficacy observed against *Salmonella typhi*. Additionally, AgO/Ag/ZnO exhibited superior inhibition of *Candida albicans* compared to the standard, along with potent anticancer activity against HCT-116 cell lines (IC_50_ value: 16.61 μg/mL) (Ramakrishnegowda et al., 2023). de Moura et al. (2022) conducted wound healing analysis, revealing an 82.7% reduction in neutrophil activity after 21 days in wounds treated with Ag-ZnO/AgO nanoparticles compared to the vehicle group (de Moura et al., 2022). A comparative literature review have also been summarized in Table 1 for the reader’s interest. Building on these findings, our present research on the ZnO/Ag/Ag_2_O_(CE)_ nanocomposite comprehensively examines its antibacterial activity, showcasing the highest zone of inhibition against *B. subtilis*, along with their antioxidant, anticancer, and wound healing activities, demonstrating 96% healing within 10 days.

**TABLE 1:** Comparative analysis of previous studies.

Compound	Antibacterial study	Antioxidant study (DPPH and reducing power assay)	Anticancer study	Wound healing	Reference
Cellulose/chitosan–Ag/Ag_2_O/ZnO (synthetic method)	*E. coli*: 15.0 mm *S. aureus*: 19.6 mm	Not reported	Not reported	Not reported	Peng et al. (2022)
Ag/Ag_2_O/ZnO nanocomposites (NCs) via Murusi peel (MP) and Kew peel (KP) (synthetic method)	Not reported	DPPH activity Ag/Ag_2_O/ZnO NCs (MP): 78.14 ± 1.04 Ag/Ag_2_O/ZnO NCs (KP): 79.18 ± 1.01	Not reported	Not reported	Aththanayaka et al. (2023)
Ag/Ag_2_O/ZnO nanocomposite (synthetic method)	7AgZn showed zones of inhibition ranging from 20 mm (*S. aureus* and *E. coli* (1)) to 13 mm (*E. coli*)	Not reported	The cytotoxicity of Ag/Ag_2_O/ZnO toward Huh-7 human liver cancer cells was investigated, yielding IC_50_ values of 112.9 μg/mL for samples prepared with 3 mL of AgNO_3_ (3AgZn) and 313 μg/mL for those prepared with 7 mL (7AgZn)	Not reported	Hezam et al. (2023)
AgO/Ag/ZnO nanocomposite (synthetic method)<	It exhibited a significant (*p* > 0.05) zone of inhibition against *E. coli* and *S. aureus*, followed by *S. typhi*, *B. subtilis*, and *C. albicans* *B. subtilis*: 21.6 ± 0.57	Not reported	AgO/Ag/ZnO nanocomposite has no such toxicity even at a concentration of 100 µg, displaying its non-poisonous property toward normal cells and anticancer potential toward HCT-116 colon cancer cells	Not reported	Ramakrishnegowda et al. (2023)
Ag-ZnO/AgO NPs (synthetic method)	Not reported	Not reported	Not reported	After 21 days, there was an 82.7% reduction in neutrophil activity in wounds treated with Ag-ZnO/AgO NPs compared to the vehicle group	de Moura et al. (2022)
ZnO/Ag/Ag_2_O_(CE)_	*B. subtilis*: 25 mm *S. aureus*: 21 mm *E. coli*: 17.5 mm	Reducing power assay: 0.573 absorbance DPPH assay: 86.554% inhibition at 1,000 μg/mL	ZnO/Ag/Ag_2_O_(CE)_ showed anticancer activity against the breast cancer cell line MCF7 with an IC_50_ value of 100.23 and 75.27% cell viability	*In vivo* treatment of wounds in an animal model with the ZnO/Ag/Ag_2_O_(CE)_ nanocomposite showed approximately 96% closure in 10 days, while the control showed ∼50% closure in 10 days	Present research

## 4 Conclusion

In conclusion, an ecofriendly method was used to fabricate stable ZnO_(CE)_ NPs and Ag/Ag_2_O_(CE)_ and ZnO/Ag/Ag_2_O_CE)_ nanocomposites using *S. hispanica* L. (commonly known as chia seeds) extract without any special capping agent. The synthesized nanocomposites were characterized by UV–visible, FTIR, SEM, and XRD techniques. The XRD analysis revealed that the crystallite sizes of ZnO_(CE)_ NPs and Ag/Ag_2_O_(CE)_ and ZnO/Ag/Ag_2_O_(CE)_ nanocomposites were 16.15 nm, 46.6 nm, and 22.42 nm, respectively. The SEM analysis showed that Ag/Ag_2_O_(CE)_ had hetero-structure particles, ZnO_(CE)_ had agglomerated nanoparticles, and ZnO/Ag/Ag_2_O_(CE)_ exhibited a spherical particle-like morphology of the composite. ZnO_(CE)_ NPs and Ag/Ag_2_O_(CE)_ and ZnO/Ag/Ag_2_O_(CE)_ nanocomposites exhibited good antibacterial activity against *S. aureus, E. coli*, and *B. subtilis*. The resistance of these nanoparticles and nanocomposites to *B. subtilis* bacterial strains was 17 mm, 16 mm, and 25.3 mm, respectively. The highest antioxidant activity, including reducing power activity and DPPH radical scavenging activity, was depicted by ZnO/Ag/Ag_2_O_(CE)_, while the lowest activity was shown by Ag/Ag_2_O_(CE)_. ZnO/Ag/Ag_2_O_(CE)_ toward the MCF7 cancer cell line yielded the highest *in vitro* anticancer activity, i.e., 75.27% inhibition at a sample concentration of 100 μg/mL. Finally, the *in vivo* treatment of wounds in an animal model with the ZnO/Ag/Ag_2_O_(CE)_ nanocomposite showed approximately 96% closure in 10 days, while the control showed ∼50% closure in 10 days. These findings emphasize the significant role of ecofriendly fabricated multifunctional ZnO/Ag/Ag_2_O_(CE)_ nanocomposites in cancer inhibition and wound healing processes.

## Data Availability

The original contributions presented in the study are included in the article/Supplementary Material, further inquiries can be directed to the corresponding authors.
