# Supplementary material for: Chia seed-mediated fabrication of ZnO/Ag/Ag2O nanocomposites: structural, antioxidant, anticancer, and wound healing studies
Source: Front Chem. 2024 Jul 11;12:1405385. doi: 10.3389/fchem.2024.1405385 (PMC11269097; doi:10.3389/fchem.2024.1405385)
Supplement: Supplementary file 1 [file DataSheet1.doc]

**Chia Seed-Mediated Fabrication of ZnO/Ag/Ag_2_O Nanocomposite: structural, antioxidant, anticancer and wound healing studies**

Aisha Rafique^a^, Fatima Amjad^b^, Muhammad Ramzan Saeed Ashraf Janjua^a^, Syed Ali Raza Naqvi^a^*, Sadaf Ul Hassan^b*^, Hanzla Abdullah^a^, Muhammad Shahid Nazir^b^, Zulfiqar Ali^b^, Abdulaziz A Alshihri^c^, Maha Abdullah Momenah^d^, Adel Abo Mansour^e^, Majed A. Bajaber^f^, Ahlam A. Alalwiat^f^

^a^Department of Chemistry, Government College University Faisalabad, 38000, Faisalabad, Pakistan

^b^Department of Chemistry, COMSATS University Islamabad, Lahore Campus, 54300, Lahore, Pakistan

^c^Department of Radiological Sciences, College of Applied Medical Sciences, King Khalid University, Abha, Saudi Arabia.

^d^ Department of Biology, College of Science, Princess Nourah bint Abdulrahman University, P.O. Box 84428, Riyadh 11671, Saudi Arabia.

^e^Department of Clinical Laboratory Sciences, College of Applied Medical Sciences, King Khalid University, Abha, Saudi Arabia.

^f^Chemistry Department, Faculty of Science, King Khalid University, P.O. Box 9004, Abha 61413, Saudi Arabia

**Corresponding Authors:** Syed Ali Raza Naqvi ([draliraza@gcuf.edu.pk](mailto:draliraza@gcuf.edu.pk)); Sadaf Ul Hassan ([sadafulhassan@cuilahore.edu.pk](mailto:sadafulhassan@cuilahore.edu.pk))

**Visual analysis**


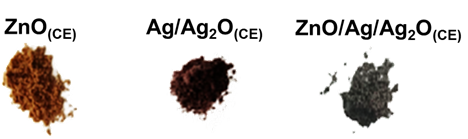


**Figure S1.** Visual analysis of Nanoparticles.

**XRD Analysis**


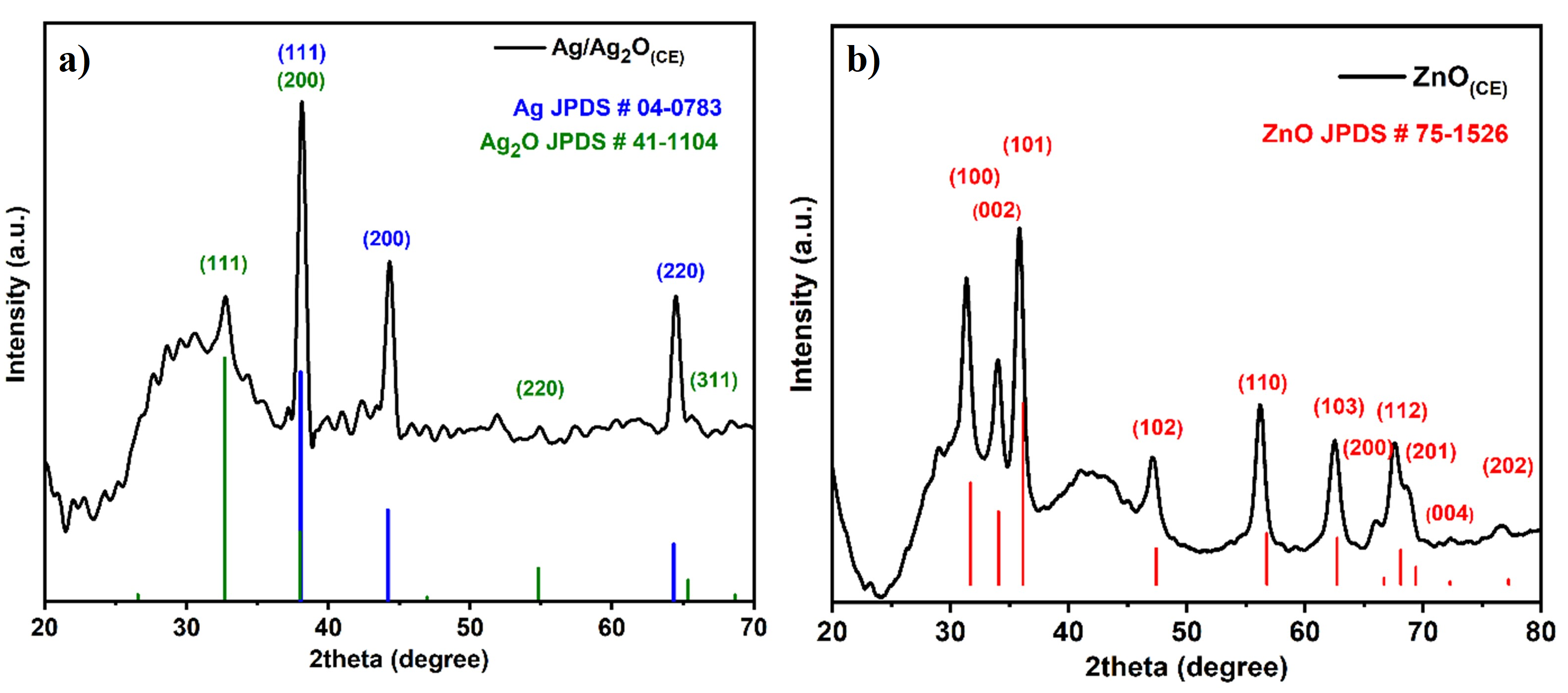


**Figure S2.** XRD analysis of (a) Ag/Ag_2_O_(CE)_ and (b) ZnO_(CE)_


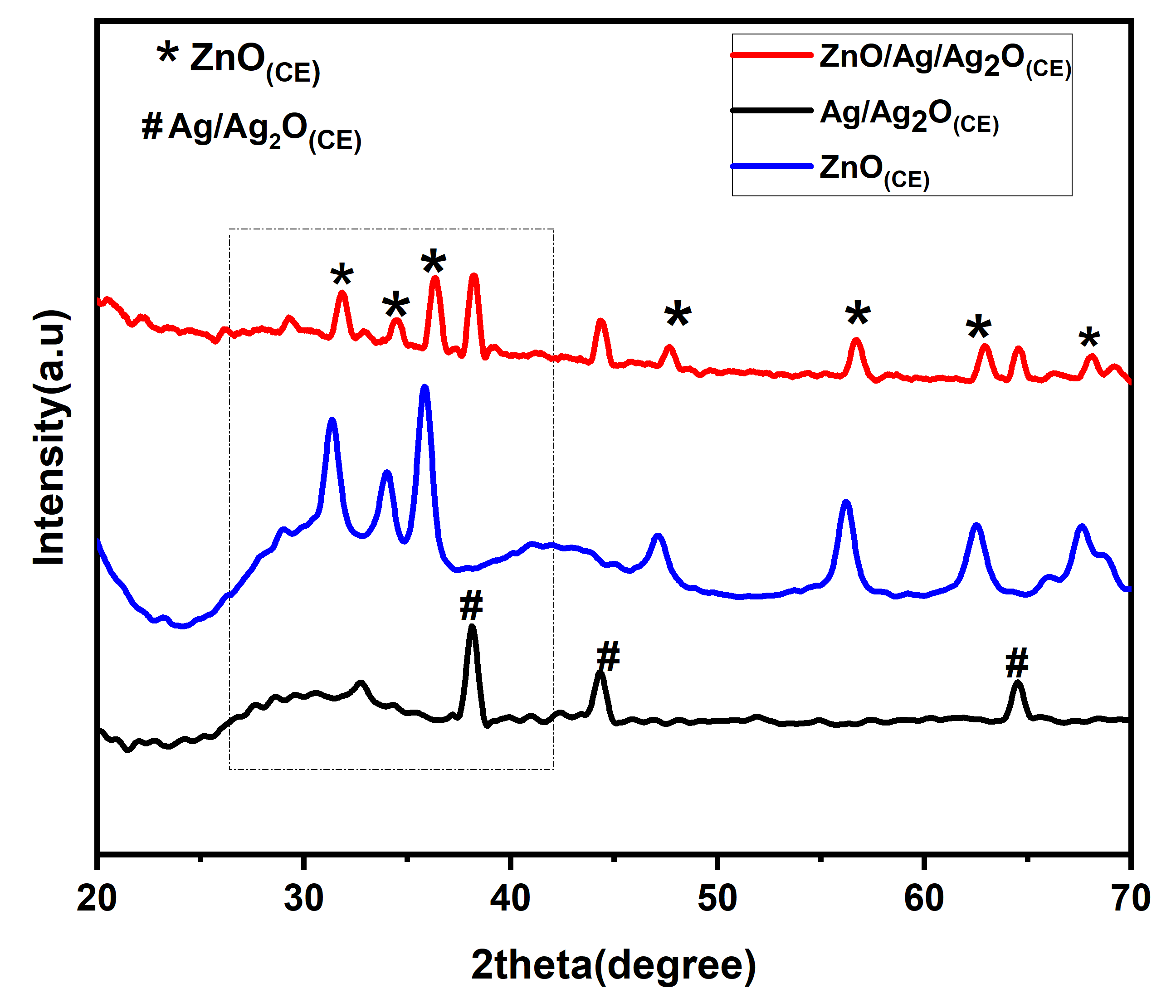


**Figure S3:** Stacked XRD graphs of ZnO_(CE)_, Ag/Ag_2_O_(CE)_, and ZnO/Ag/Ag_2_O_(CE)_

**UV-Visible Studies**

**
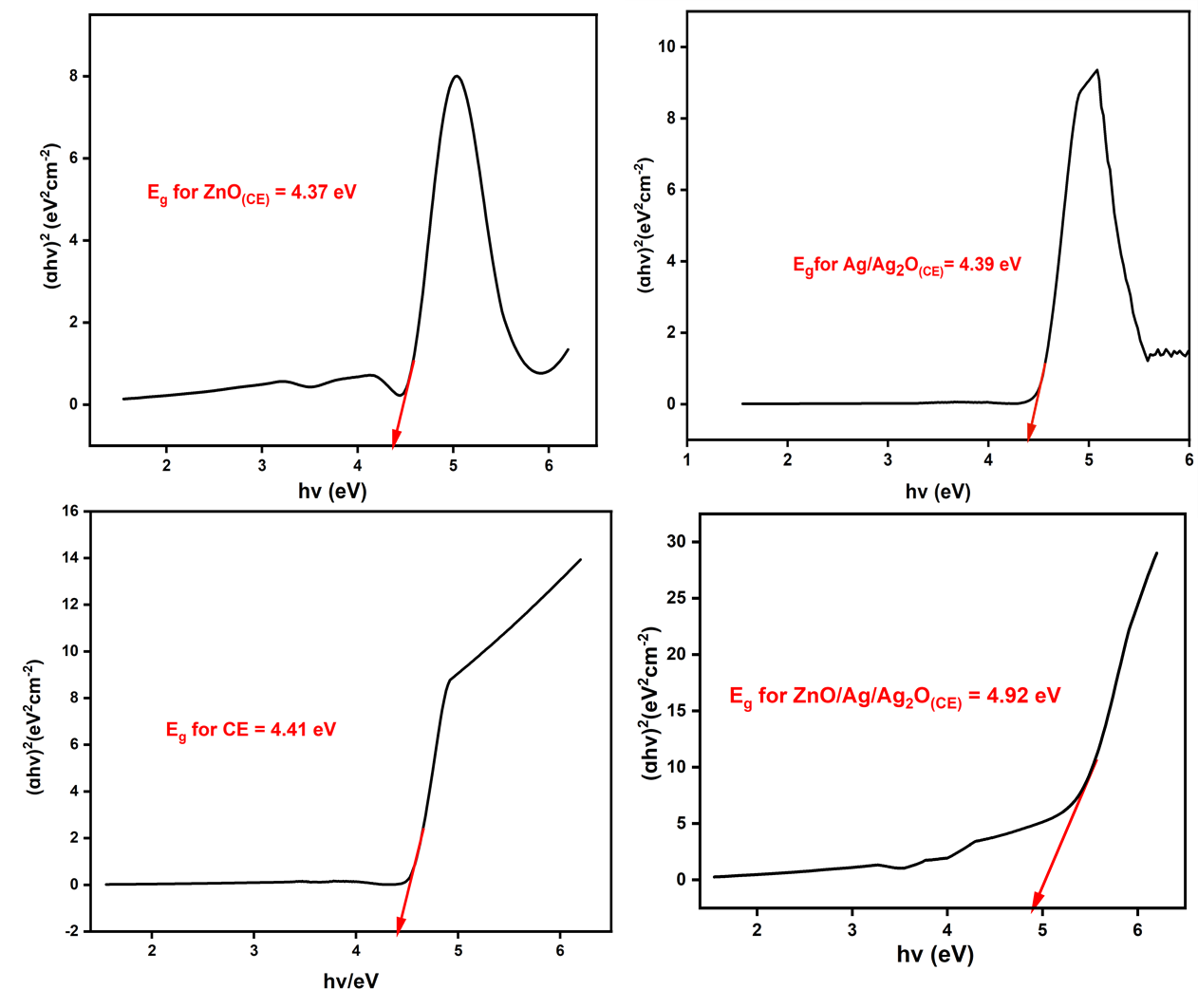
**

**Figure S4.** Tauc’s Plot analysis for band gap energy calculations of the prepared nanocomposites.

**EDX Analysis**

**
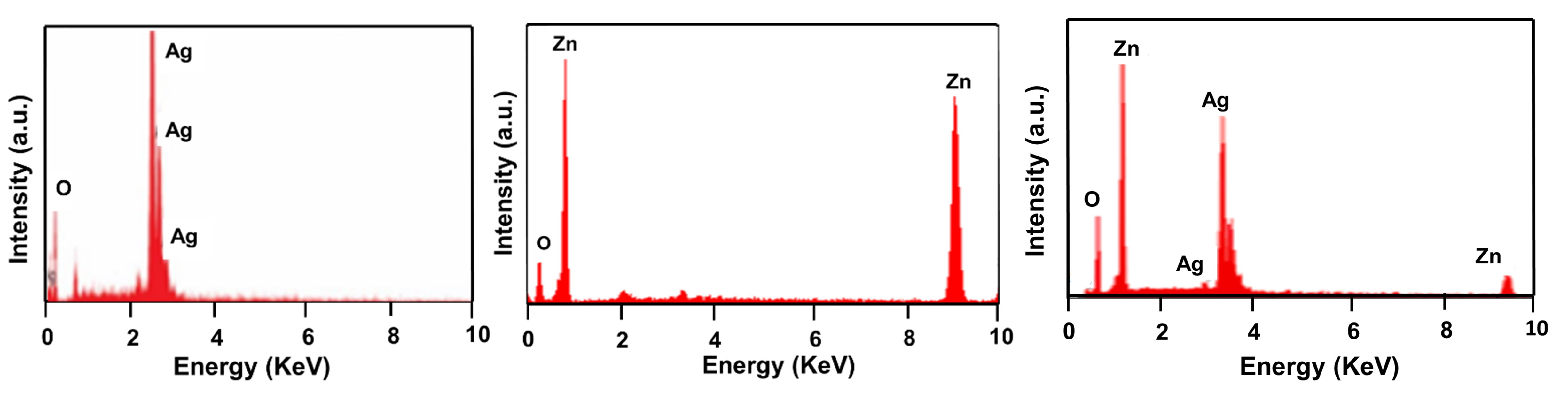
Figure S5.** EDX analysis of Ag/Ag_2_O_(CE)_, ZnO_(CE)_ nanoparticles and ZnO/Ag/Ag_2_O _(CE)_ nanocomposite.


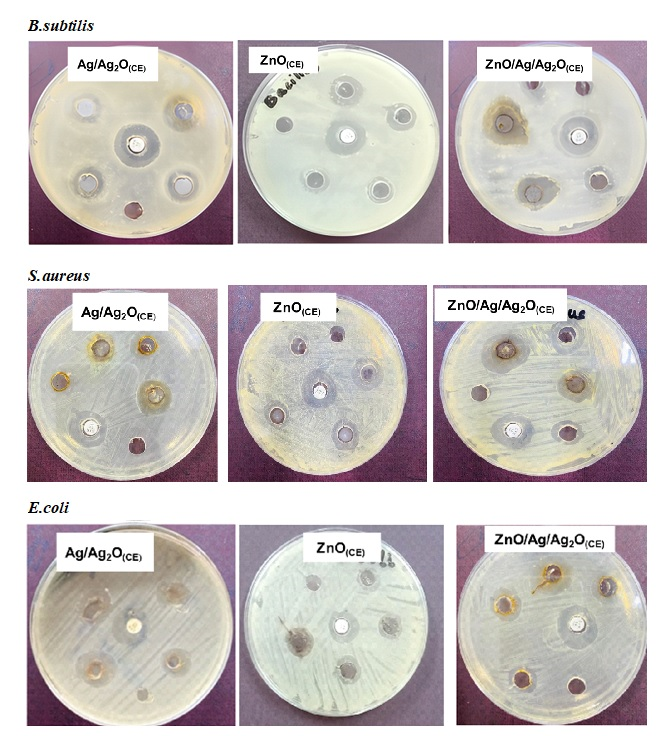


**Figure S6.** Antibacterial analysis of nanocomposites using different bacterial strains.

**Wound Healing Studies
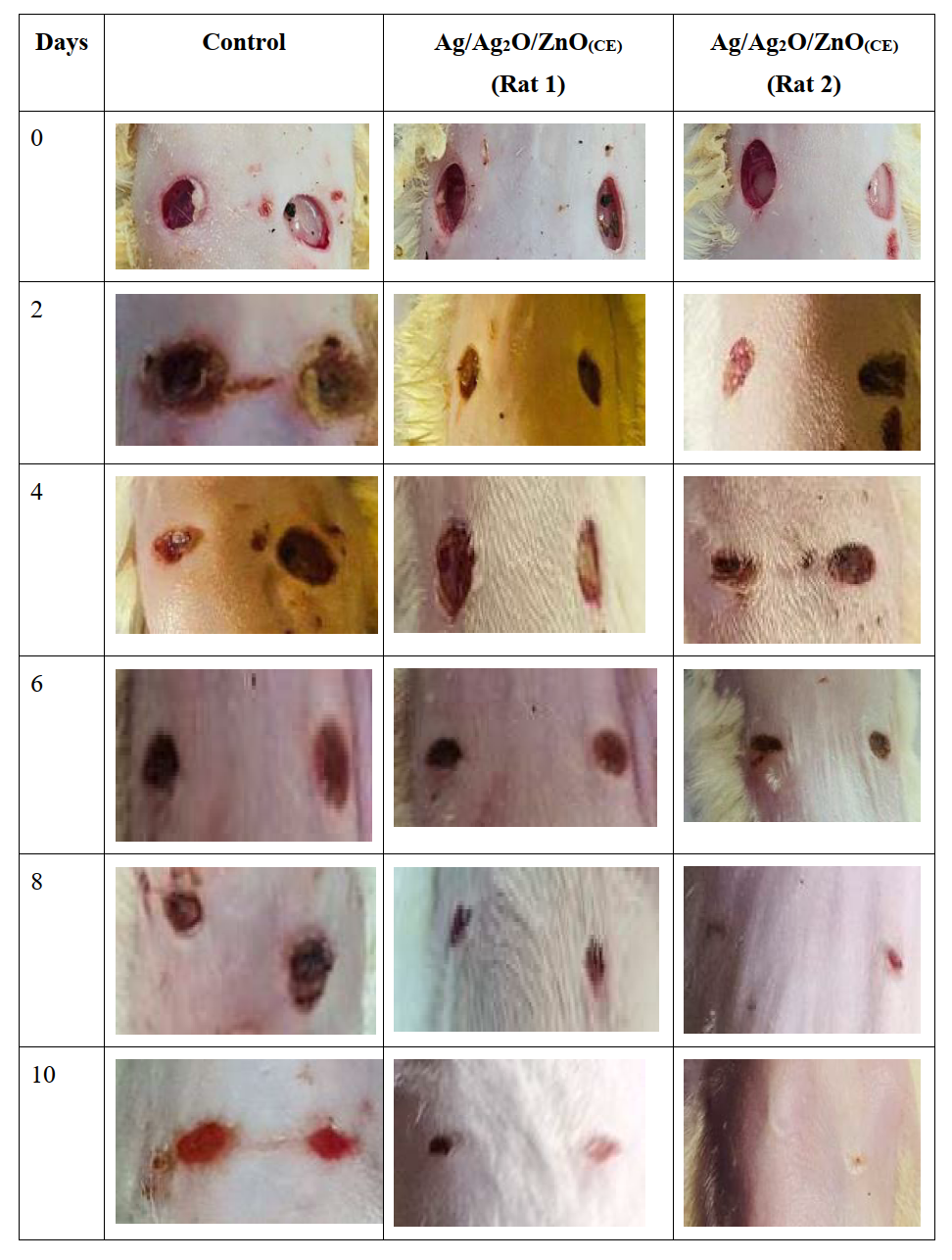
** **Figure S7:** Complete wound healing duration in rats.
